# Desloratadine Therapy Improves Allergic Rhinitis Symptoms in Latin American Children Aged 6 to 12 Years

**DOI:** 10.1097/WOX.0b013e31819cdfdb

**Published:** 2009-04-15

**Authors:** Paolo Tassinari, Nelson R Suárez, Jorge Centeno, Janina Vergara Velásquez, Héctor Aguirre-Mariscal, Sandra N González Díaz, Alfredo Fernández de Córdova Jerves

**Affiliations:** 1 Instituto de Immunología, Universidad Central de Venezuela; 2 Policlínica Metropolitana, Caracas, Venezuela; 3 Universidad Peruana Cayetano Heredia, Lima, Perú; 4 Centro Especializado San Fernando, Panamá City, Panamá; 5 Hospital Angeles Pedregal, Mexico City; 6 Allergy and Clinical Immunology Department, Hospital Universitario, Monterrey, Mexico; 7 Hospital Universitario del Río, Universidad del Azuay, Cuenca, Ecuador; 8 Universidad Central de Venezuela, Apartado 47114, Los Chaguaramos, Caracas 1040A, Venezuela

**Keywords:** allergic rhinitis, children, congestion, desloratadine

## Abstract

**Objective**

To determine the effectiveness of desloratadine syrup in relieving symptoms of allergic rhinitis (AR) among children in Latin America.

**Methods**

In an open-label trial conducted in 5 Latin American countries, 455 children aged 6 to 12 years with seasonal or perennial AR were treated with desloratadine syrup 2.5 mg/d for 6 weeks. Thirty percent of subjects were concomitantly taking corticosteroids, and 21.3% had a history of asthma. Efficacy was measured by improvement in the Total Symptom Severity 4 questionnaire and decrease in severity of individual nasal symptoms of congestion, rhinorrhea, pruritus, and sneezing. Physicians and subjects' caregivers rated symptom improvement in a separate assessment at final visit.

**Results**

Treatment with desloratadine led to a significant decrease in mean Total Symptom Severity 4 score, from 7.54 at baseline to 1.96 at study end (*P *< 0.0001), and in individual symptom scores, including congestion (*P *< 0.0001 for all). Similar improvements were found in groups receiving desloratadine monotherapy and desloratadine plus corticosteroids. Allergic rhinitis symptoms were rated "better" or "much better" by 94% of caregivers. Incidence of adverse events was 6%.

**Conclusions**

Desloratadine, with or without concomitant corticosteroids, was efficacious and safe in the treatment of AR in this group of Latin American children.

## 

Allergic rhinitis (AR) symptoms can reduce children's performance in school, and certain medications used to treat AR can exacerbate this problem [1,2]. Fatigue, cognitive impairment, irritability, inattentiveness, difficulty concentrating, behavioral problems, poor test performance, and absenteeism are among the causes of poor school performance [3-5]. A recent study of adolescents in the United Kingdom found that students who had AR were 40% more likely to drop a grade in their school examinations between winter and summer than healthy students,[6] and Sundberg et al [7] noted that severe nasal symptoms were associated with lower grades in Swedish adolescents. The American Academy of Allergy, Asthma & Immunology has estimated that children in the United States lose 2 million school days a year as a result of AR [8].

The guidelines issued by the Allergic Rhinitis and its Impact on Asthma Workshop Group do not recommend the use of first-generation sedating antihistamines--such as diphenhydramine, chlorpheniramine, and brompheniramine--for the treatment of AR because, in part, of their marked sedative effects [9]. The US Food and Drug Administration Nonprescription Drugs Advisory Committee recently recommended that over-the-counter cold and cough medications, some of which contain first-generation antihistamines, not be used in children aged 2 to 5 years. In a review of its records, the US Food and Drug Administration found 69 reports of deaths associated with antihistamine medicines containing diphenhydramine, chlorpheniramine, or brompheniramine [10].

Nonsedating second-generation antihistamines are recommended for use in adults and adolescents [9,11]. The Allergic Rhinitis and its Impact on Asthma Workshop Group and the European Academy of Allergology and Clinical Immunology guidelines for pediatric patients are generally similar to those for older patients, but they are less specific when discussing the treatment of children [9,11,12]. The American Academy of Allergy, Asthma & Immunology guidelines recommend oral antihistamines as a first-line therapy for children with AR [13].

In controlled clinical trials, second-generation antihistamines have significantly reduced nasal congestion in subjects 12 years or older [14-17]. Nasal congestion is often the only presenting symptom of AR in children. Desloratadine, a nonsedating second-generation antihistamine, has demonstrated efficacy for the treatment of AR symptoms in adults and adolescents,[15,18,19] and results of an uncontrolled efficacy trial suggest that it is effective in children aged 6 to 12 years [20]. Randomized, double-blind, placebo-controlled safety studies have shown that desloratadine is safe and well tolerated in children as young as 6 months [21-24].

Although the prevalence of pediatric AR in Latin America has not been extensively studied, AR may affect 40% or more children in the United States by the age of 6 years [25,26]. Data drawn from patient questionnaires and physician examinations in North America and western Europe suggest that seasonal AR affects 15% to 22% of adolescents. In some studies, perennial AR is estimated to affect only about 1% to 8% [9]. In Latin American study centers (defined as a city or a specified geographic area) participating in the International Study of Asthma and Allergies in Childhood, the percentage of children who reported AR symptoms during the previous 12 months ranged from 21% in Panama to 42% in Argentina among 6- to 7-year olds, and from 16% in Chile to 67% in Paraguay among 13- to 14-year olds [27]. A trial conducted in 13- to 14-year olds in Brazil during 2002 found that 29% were current AR sufferers, and that the monthly prevalence of AR peaked during the winter months of June, July, and August [9].

Because the frequency of AR varies widely among countries primarily because of environmental factors, determining patient response to medications in different geographic areas is important [9]. The purpose of this study was to determine the effectiveness of desloratadine in providing relief of symptoms of AR among children in 5 Latin American countries.

## Materials and methods

### Patient Population

An open-label, observational, multicenter study was conducted in pediatric subjects from approximately 100 health care centers in Mexico, Peru, Venezuela, Ecuador, and Panama from October 1, 2004, through November 30, 2005. Subjects aged 6 to 12 years who had a physician-confirmed diagnosis of either seasonal or perennial AR were included. The individual diagnosis, initiation of treatment, and the necessary monitoring and follow-up visits depended on the underlying medical condition and the treatment prescribed, according to the physician's routine practice. If the subject or his/her guardian agreed to participate in the study, treatment with desloratadine was initiated, and clinical data were prospectively collected.

At the enrollment (baseline) visit, investigators collected demographic data, including a personal and/or family history of other atopic/allergic conditions and a list of AR triggers; the severity of nasal AR symptoms was determined using a questionnaire. Eligible subjects took desloratadine syrup 2.5 mg/d (5 mL) for 6 weeks after the baseline visit. Concomitant corticosteroid therapy (inhaled, oral, or intranasal) was permitted on an as-needed basis during the study period.

Subjects returned for a second (final) study visit at the end of the treatment period. The use of all concomitant medications, including corticosteroids, was documented at this visit, and efficacy assessments were made. Any adverse events (AEs), regardless of whether they were related to study medication, were recorded at this time as well.

Study protocol and informed consent forms were approved by the institutional review board for each medical center. The study was conducted in compliance with Good Clinical Practice guidelines and under the principles of the Helsinki Declaration. Reports of all serious AEs were communicated as soon as possible to the appropriate institutional review board and/or reported in accordance with local laws and regulations.

### Efficacy Measures

**Figure 1:**
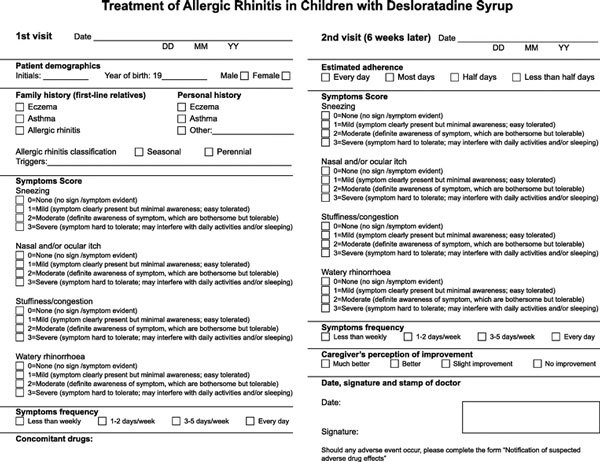
**The Total Symptom Severity 4 questionnaire**.

Efficacy was measured using the Total Symptom Severity (TSS4) questionnaire (Figure 1), which rates the severity of nasal congestion, rhinorrhea, nasal pruritus, and sneezing using a 4-point scale: 0, absent (no signs or symptoms evident); 1, mild (signs and symptoms present, but minimal awareness and easily tolerated); 2, moderate (signs and symptoms definitely present and bothersome, but tolerable); and 3, severe (signs and symptoms hard to tolerate and may cause interference with activities of daily living or sleeping). The questionnaire was completed by the investigators at the baseline and final visits. Scores from the 2 assessments of the 4 symptoms were added to obtain the total TSS4 score. The TSS4 scores recorded at the baseline and final visits were compared using a paired t test.

At the final visit, subjects, caregivers, and physicians separately rated improvements from baseline in nasal symptoms. Subjects were asked to rate their perception of symptomatic improvement as none, mild, better, or much better, and physicians rated each subject's symptoms as worse, no change, or better after treatment.

## Results

### Demographics

The study included 455 children aged 6 to 12 years (mean age, 8.36 ± 2.02 years) with either seasonal or perennial AR. Subjects were residents of Mexico (29.1%, n = 132), Peru (24.2%, n = 110), Venezuela (22.7%, n = 103), Ecuador (13.7%, n = 62), or Panama (10.3%, n = 48). The contribution of subjects to the sample size was prespecified based on the capacity or resources of the medical centers of each participating country. Sixty-two percent (n = 282) of subjects were boys.

Perennial AR was observed in 61% of subjects (n = 277), seasonal AR in 30% (n = 138), and both in 0.2% (n = 1). Thirty-nine subjects did not state which type of AR they had. Most subjects experienced frequent AR symptoms: 45% (n = 204) reported that these symptoms affected them daily, and 23% (n = 105) were affected 3 to 5 days a week. Ninety-two percent (n = 417) of subjects reported triggers for their AR. House dust, pet dander, and pollen, either alone or in combination, were the most frequently cited allergens, reported by 80% (n = 334), 24% (n = 101), and 19% (n = 79) of subjects, respectively. Ninety-nine percent of subjects (n = 447) had congestion, and 96% (n = 436) had sneezing.

Nearly 10% of subjects (n = 45) reported concomitant eczema, and 21.3% (n = 97) reported concomitant asthma (Table 1). Eighty-six percent of subjects (n = 393) had a family history of AR (42.2%, n = 192), asthma (11.0%, n = 50), eczema (3.7%, n = 17), or a combination (29.5%, n = 134) (Table 2). Thirty percent (n = 136) of subjects took concomitant inhaled, oral, or intranasal corticosteroids.

**Table 1:** Personal History of Concomitant Disease

Disease	n	%
Eczema	45	9.9
Asthma	97	21.3
Eczema and asthma	24	5.3
Other*	29	6.4
Asthma and other	12	2.6
Eczema, asthma, and other	3	0.7
Total with history	210	46.2
No history	245	53.8
Total	455	100.0

*Urticaria, conjunctivitis, adenoiditis, pharyngitis, sinusitis, other inflammatory processes, or unspecified.

**Table 2:** Family History of Allergic Disease

Disease	n	%
Eczema	17	3.7
Asthma	50	11.0
Allergic rhinitis	192	42.2
Eczema and asthma	7	1.5
Eczema and rhinitis	25	5.5
Asthma and rhinitis	79	17.4
Eczema, asthma, and rhinitis	23	5.1
Total with history	393	86.4
No history	62	13.6
Total	455	100.0

**Figure 2:**
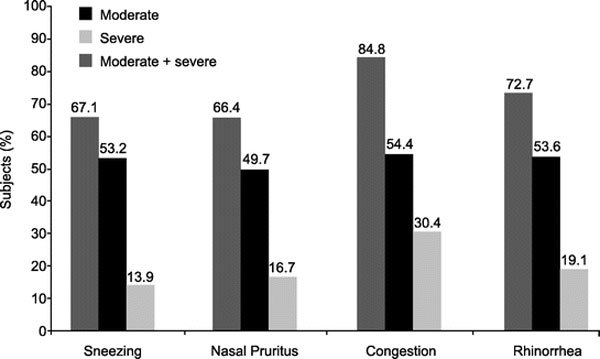
**Percentage of children with moderate or severe symptoms of AR at baseline**.

Most subjects (95%) returned for follow-up between 28 and 77 days. At baseline, slightly more than half of subjects rated sneezing, nasal itching, congestion, and rhinorrhea as moderate or severe (Figure 2).

### Efficacy

**Figure 3:**
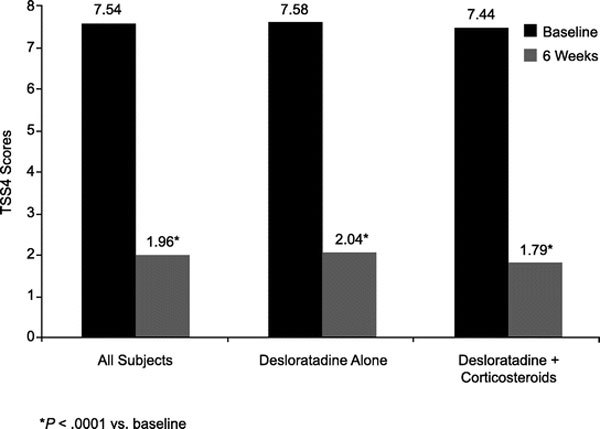
**The TSS4 scores at baseline and after 6 weeks of desloratadine therapy in all subjects and according to the use of concomitant corticosteroid therapy**.

**Figure 4:**
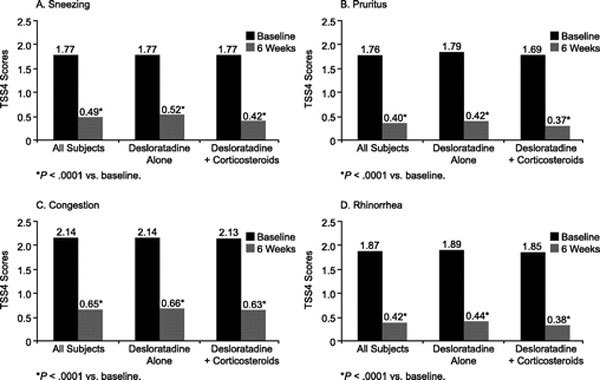
**Changes in individual symptom scores in all subjects and according to the use of concomitant corticosteroid therapy**.

For all subjects, the TSS4 score decreased significantly from a mean of 7.54 at baseline to 1.96 at the final visit (*P *< 0.0001). Significant reductions were observed in TSS4 scores from baseline in subjects taking desloratadine monotherapy (from 7.58 to 2.04, *P *< 0.0001) or desloratadine with concomitant corticosteroids (from 7.44 to 1.79, *P *< 0.0001) (Figure 3). Subjects treated with desloratadine monotherapy had a reduction in mean nasal congestion scores from 2.14 to 0.66 (*P *< 0.0001), which was comparable to the reduction seen in subjects treated with desloratadine plus corticoste-roids (from 2.13 to 0.63, *P *< 0.0001). Decreases in the other individual symptom scores were also significant (*P *< 0.0001) for all subjects (Figure 4).

**Figure 5:**
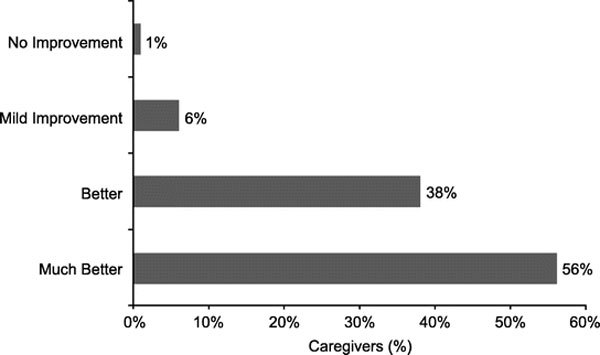
**Caregivers' perceptions of improvement**.

After desloratadine therapy, 94% (n = 421) of caregivers rated the subjects' overall symptoms as "better" or "much better" (Figure 5). Physicians gave ratings of "better" for overall symptoms in 79% (n = 337) of subjects, for sneezing in 86% (n = 388), for nasal pruritus in 87% (n = 393), for congestion in 89% (n = 403), and for rhinorrhea in 89% (n = 404).

### Safety

Six percent of subjects (n = 25) reported a total of 33 AEs, the most frequent being headache (7/33; 21%). None of the AEs were serious. Most AEs with an identifiable cause were caused by coexisting disease (n = 13), and AEs were considered likely related to study medication in only 4 subjects. Five subjects discontinued the study because of AEs: epistaxis (n = 2), headache/drowsiness (n = 1), gastric intolerance (n = 1), or rash with pruritus (n = 1).

## Discussion

Worldwide studies indicate that although AR is common in young schoolchildren, the prevalence is greater in some geographic regions, such as South America and countries on the Pacific Rim [9,27,28]. A study of Brazilian adolescents found that prevalence of diagnosed AR increased by 37% from 1996 to 2002,[28] a rise that is consistent with previous reports in other countries [9,29,30].

Allergic rhinitis symptoms can impair a child's school performance and affect behavior influenced by sleep disruption. Some of these impairments may be related to nasal congestion, which both adults and children consider the most bothersome symptom of AR [31]. In a large survey of adults in the United States (N = 2355) who had AR or cared for a child with AR, 61% of caregivers reported that nasal congestion caused by AR affected their child's performance at school; the most common effects were poor productivity or inability to concentrate [31]. The same survey reported that nasal congestion caused night-time awakening and difficulty falling asleep in nearly 50% of children. Another large survey (N = 4927) found that adults who consistently experienced nighttime congestion related to AR were twice as likely to report habitual snoring, chronic excessive daytime sleepiness, and non-restorative sleep [32]. Therefore, medications used to treat AR should be nonsedating and not cause additional cognitive impairments.

In the present study conducted in 5 Latin American countries, most of the children enrolled (61%) had perennial AR. Subjects identified house dust and pet dander as the most frequent triggers of their symptoms, and 21% had a history of asthma. Indoor allergens also exacerbate asthma, and this result underscores the need for a comprehensive therapeutic strategy that includes environmental control measures in addition to an effective therapeutic agent.

The results of this study indicate that desloratadine is an efficacious and safe treatment choice for Latin American children with perennial or seasonal AR. Desloratadine therapy significantly reduced nasal congestion in all subjects (*P *< 0.0001); similar decreases were noted for rhinorrhea, nasal pruritus, and sneezing. Nearly all of the caregivers rated symptoms of AR as "better" or "much better" at the end of treatment. The response in the overall patient population and groups treated with desloratadine alone or desloratadine plus corticosteroids was comparable (*P *< 0.0001 for all groups), indicating that concomitant corticosteroid therapy did not have an additional therapeutic or detrimental effect on AR symptoms.

Good tolerability in a pediatric population predominantly affected by perennial AR is important because unlike seasonal AR, which can be treated with a short course of therapy during the pollen season, perennial AR generally requires long-term treatment [10]. Subjects in this trial reported a low incidence of all AEs (6%), and desloratadine was safe and well tolerated.

The objective of this study was to evaluate improvement in only the symptoms of AR. For this reason, the conclusion--that there is no difference in outcome whether desloratadine is used alone or with intranasal corticosteroids--should be considered with some caution. Additional studies are needed before deciding that Latin American children with AR can be treated as effectively with desloratadine monotherapy as with combination therapy.

The results of this open-label, observational, multi-center study show that symptoms of AR, including nasal congestion, were improved with desloratadine therapy in this group of pediatric subjects. Desloratadine is an efficacious and safe choice for physicians in Latin America to prescribe for children with perennial or seasonal AR.

## Note

Supported by Schering-Plough.

Presented at the 2007 American Academy of Allergy, Asthma, & Immunology Annual Meeting; February 23-27, 2007; San Diego, CA. Poster 248.
